# Humoral immunity against SARS-CoV-2 evoked by heterologous vaccination groups using the CoronaVac (Sinovac) and BNT162b2 (Pfizer/BioNTech) vaccines in Chile

**DOI:** 10.3389/fpubh.2023.1229045

**Published:** 2023-08-24

**Authors:** Diego A. Díaz-Dinamarca, Pablo Díaz, Gisselle Barra, Rodrigo Puentes, Loredana Arata, Jonnathan Grossolli, Boris Riveros-Rodriguez, Luis Ardiles, Julio Santelises, Valeria Vasquez-Saez, Daniel F. Escobar, Daniel Soto, Cecilia Canales, Janepsy Díaz, Liliana Lamperti, Daniela Castillo, Mychel Urra, Felipe Zuñiga, Valeska Ormazabal, Estefanía Nova-Lamperti, Rosana Benítez, Alejandra Rivera, Claudia P. Cortes, María Teresa Valenzuela, Heriberto E. García-Escorza, Abel E. Vasquez

**Affiliations:** ^1^Sección de Biotecnología, Departamento Agencia Nacional de Dispositivos Médicos, Innovación y Desarrollo, Instituto de Salud Pública de Chile, Santiago, Chile; ^2^Sección gestión de la información, Departamento Agencia Nacional de Dispositivos Médicos, Innovación y Desarrollo, Instituto de Salud Pública de Chile, Santiago, Chile; ^3^Tecnología Medica, Facultad de Medicina, Clínica Alemana-Universidad del Desarrollo, Universidad del Desarrollo, Santiago, Chile; ^4^Departamento de Bioquímica Clínica e Inmunología, Facultad de Farmacia, Universidad de Concepción, Concepción, Chile; ^5^Unidad de investigación Clínica, Clínica Dávila, Santiago, Chile; ^6^Millennium Institute on Immunology and Immunotherapy, Santiago, Chile; ^7^Facultad de Medicina, Universidad de Chile, Santiago, Chile; ^8^Clínica Santa María, Santiago, Chile; ^9^Facultad de Medicina, Universidad de Los Andes, Las Condes, Chile; ^10^Instituto de Salud Pública de Chile, Santiago, Chile; ^11^Departamento de Investigación, Postgrado y Educación Continua (DIPEC), Facultad de Ciencias de la Salud, Universidad del Alba, Santiago, Chile

**Keywords:** SARS-CoV-2, Chilean vaccination, SARS-CoV-2 neutralizing antibodies, COVID19, BNT162b2 (Pfizer-BioNTech), CoronaVac vaccine, heterologous vaccination, immunization schedules

## Abstract

**Introduction:**

Severe acute respiratory syndrome virus 2 (SARS-CoV-2) has caused over million deaths worldwide, with more than 61,000 deaths in Chile. The Chilean government has implemented a vaccination program against SARS-CoV-2, with over 17.7 million people receiving a complete vaccination scheme. The final target is 18 million individuals. The most common vaccines used in Chile are CoronaVac (Sinovac) and BNT162b2 (Pfizer-Biotech). Given the global need for vaccine boosters to combat the impact of emerging virus variants, studying the immune response to SARS-CoV-2 is crucial. In this study, we characterize the humoral immune response in inoculated volunteers from Chile who received vaccination schemes consisting of two doses of CoronaVac [CoronaVac (2x)], two doses of CoronaVac plus one dose of BNT162b2 [CoronaVac (2x) + BNT162b2 (1x)], and three doses of BNT162b2 [BNT162b2 (3x)].

**Methods:**

We recruited 469 participants from Clínica Dávila in Santiago and the Health Center Víctor Manuel Fernández in the city of Concepción, Chile. Additionally, we included participants who had recovered from COVID-19 but were not vaccinated (RCN). We analyzed antibodies, including anti-N, anti-S1-RBD, and neutralizing antibodies against SARS-CoV-2.

**Results:**

We found that antibodies against the SARS-CoV-2 nucleoprotein were significantly higher in the CoronaVac (2x) and RCN groups compared to the CoronaVac (2x) + BNT162b2 (1x) or BNT162b2 (3x) groups. However, the CoronaVac (2x) + BNT162b2 (1x) and BNT162b2 (3x) groups exhibited a higher concentration of S1-RBD antibodies than the CoronaVac (2x) group and RCN group. There were no significant differences in S1-RBD antibody titers between the CoronaVac (2x) + BNT162b2 (1x) and BNT162b2 (3x) groups. Finally, the group immunized with BNT162b2 (3x) had higher levels of neutralizing antibodies compared to the RCN group, as well as the CoronaVac (2x) and CoronaVac (2x) + BNT162b2 (1x) groups.

**Discussion:**

These findings suggest that vaccination induces the secretion of antibodies against SARS-CoV-2, and a booster dose of BNT162b2 is necessary to generate a protective immune response. In the current state of the pandemic, these data support the Ministry of Health of the Government of Chile’s decision to promote heterologous vaccination as they indicate that a significant portion of the Chilean population has neutralizing antibodies against SARS-CoV-2.

## Introduction

The severe acute respiratory syndrome coronavirus 2 (SARS-CoV-2) virus – responsible for coronavirus disease 2019 (COVID-19) – was initially identified in Wuhan, Hubei Province, China, in December 2019 (1). On March 11, 2020, the World Health Organization (WHO) declared COVID-19 a global pandemic (2). Beginning soon after identifying this viral disease, there has been an unprecedented scientific effort to develop therapies and treatments to contain the pandemic. However, due to the lack of efficient treatment, vaccines emerged as the primary strategy to combat the global situation (3, 4), leading to the rapid development of vaccines (5).

More than 80 vaccine candidates have been developed against SARS-CoV-2 (6). In Chile, CoronaVac (Sinovac) and BNT162b2 (Pfizer/BioNTech) were the two most commonly used vaccines for the initial two doses in 2021 (7, 8). These vaccines employ different manufacturing technologies. CoronaVac is a chemically inactivated whole virus vaccine (7, 9), whereas BNT162b2 is an mRNA-based vaccine that encodes the Spike protein and is packaged in liposomes (10, 11). BNT162b2 vaccines elicit a protective immune response by generating neutralizing antibodies against the Spike protein of SARS-CoV-2. On the other hand, CoronaVac induces a broad range of antibodies against both the nucleoprotein and Spike protein (12). However, it has been observed that the activity of neutralizing antibodies, whether generated through vaccination or infection, declines over time. Consequently, the administration of booster doses with the same or different vaccines against SARS-CoV-2 has become necessary (13).

In Chile, the government recommended administering a third booster dose to the population 6 months after the second immunization (14). The primary booster was the BNT162b2 vaccine, resulting in a combination of vaccines being administered to the population. Most of the Chilean population received two doses of CoronaVac followed by one dose of BNT162b2, while a smaller group received three doses of BNT162b2 (15–17). The heterologous use of vaccines in Chile has provided an opportunity to study the humoral immune response in the population, which may help guide future booster strategies, if necessary. In this study, we present the results of the humoral immunological evaluation following different vaccine schedules in the Chilean population. A total of 457 volunteers from major cities, including Santiago and Concepción, were recruited. These volunteers received immunizations with CoronaVac, BNT162b2, or a combination of both vaccines. We assessed the production of antibodies against the *N* and RBD proteins of SARS-CoV-2 and the presence of neutralizing antibodies against viral infection.

## Methods

### Donor recruitment and sample collection.

We enrolled donors who had received two doses of CoronaVac [CoronaVac (2x)], two doses of CoronaVac followed by one dose of BNT162b2 [CoronaVac (2x) + BNT162b2 (1x)], or three doses of BNT162b2 [BNT162b2 (3x)]. It is important to note that these vaccination schemes were developed against the original strain of SARS-CoV-2 (Wuhan) when administered in Chile. In addition, we included samples from individuals who had previously been infected with COVID-19 and subsequently recovered without receiving any vaccination (RCN). We also collected pre-pandemic serum samples for comparison. The samples were obtained from Clínica Dávila, Clínica Santa María in Santiago, Chile, and the Víctor Manuel Fernández Health Family Center in the city of Concepción, Chile. We recorded the medical histories of all patients, which are summarized in Table 1.

**Table 1:** Vaccination scheme, gender, age range, and morbidity backgrounds of individuals included in this study.

Variable	Recovered non vaccinated	CoronaVac (2x)	CoronaVac (2x) + BNT162b2 (1x)	BNT162b2 (3x)
Gender male	13 (48.1%)	14 (26.4%)	70 (40%)	55 (39%)
Gender female	14 (51.9%)	39 (73.6%)	105 (60%)	73 (61%)
Hypertension	2 (7.4%)	5 (9.1%)	15 (8.6%)	7 (5.5%)
Weight (mean)	76.6 Kg	71.2 Kg	73.4 Kg	69.1 Kg
COVID +	27 (100%)	0 (0%)	0 (0%)	0 (0%)
BMI—normal	9 (33.3%)	25 (47.2%)	70 (40%)	80 (62.5%)
BMI—overweight	13 (48.1%)	19 (35.9%)	66 (37.7%)	38 (29.7%)
BMI—obese	5 (18.6%)	8 (15.1%)	39 (22.3%)	8 (6.3%)
Age range 18–19	0 (0%)	0 (0%)	1 (0.6%)	50 (39.1%)
Age range 20–29	2 (7.4%)	12 (22.6%)	49 (28%)	46 (35.9%)
Age range 30–39	6 (22.2%)	18 (34.0%)	49 (28%)	6 (4.7%)
Age range 40–49	12 (44.4%)	11 (20.8%)	41 (23.4%)	5 (3.9%)
Age range 50–59	4 (14.8%)	8 (15.1%)	20 (11.42%)	12 (9.4%)
Age range ≥ 60	3 (11.1%)	4 (7.5%)	15 (8.6%)	9 (7%)
Total	27 (100%)	53 (100%)	175 (100%)	128 (100%)

The patients were categorized into two groups: post-infected and vaccinated. The main inclusion criteria for both groups were individuals between 18 and 80 years old and a negative result on a PCR test for SARS-CoV-2 conducted at the time of sample collection. The main exclusion criteria included individuals under 18 or over 80 years old, pregnant women, individuals with morbid obesity, immunosuppression, or symptoms associated with COVID-19.

The post-infection group consisted of individuals who had recovered from COVID-19 infection within a maximum time frame of 8 months post-infection and had previously required hospitalization. Individuals in this group who had received a SARS-CoV-2 vaccine or had a history of reinfection by COVID-19 were excluded.

The vaccinated group included individuals who had completed their immunization schedule at least 21 days before sample collection and no more than 6 months before the collection. In addition, individuals with a history of previous infections (as declared by the donor) were excluded from this group.

A nasopharyngeal swab (NPS) sample and a minimum of 5 mL of blood were collected from each donor. The NPS samples were obtained following the respective guidelines of Clínica Dávila, Clínica Santa María, and Víctor Manuel Fernández Health Family Center. The age distribution of the vaccination schemes is presented in Table 1. A total of 356 donors were included in the study. After applying a documentary filter, 175 donors who had received the CoronaVac (2x) + BNT162b2 combination remained, and 128 donors received the triple BNT162b2 scheme, excluding all individuals with positive IgG against *N* in this group. Subsequently, IgG against *N* CLIA Chemiluminescence immunoassay was performed, resulting in a viable sample size of 128 for the triple BNT162b2 group. All participants received their last vaccine dose at least 3 months before the blood sample collection.

Additionally, we identified 53 donors who had received the double CoronaVac vaccination at Clínica Santa María. A total of 27 samples were obtained from the RCN group, consisting of individuals who had recovered from COVID-19 without being vaccinated, and 40 pre-pandemic serum samples were included in the study. Samples were collected with authorization from the local ethics committee, and all participants provided informed consent by declaring and signing it.

### Serum preparation

Venous blood was collected using a vacuum blood collection tube with clot activation and separating gel (BD Vacutainer) to obtain serum. The tube was kept at room temperature for at least 30 min to allow the blood to clot. Subsequently, it was centrifuged at 750 *xg* for 10 min at 4°C. A total of 2 mL of supernatant serum was collected and divided into two 1 mL cryotubes. The cryotubes were then stored at −20°C for further analysis.

### Antibody detection

Immunoglobulin G against protein N, S1-RBD, and neutralizing antibodies were detected using a chemiluminescent immunoassay (CLIA) with the SNIBE commercial kit (Snibe Diagnostic, cat. 130,219,015 M, 130219017 M, and 130,219,027 M, respectively). In summary, 300 μL of serum was aliquoted into cryotubes and analyzed using the Maglumi X8 CLIA detector following the manufacturer’s instructions. Based on the CLIA commercial kit analysis (Anti-IgG-N and S1-RBD), any sample with an arbitrary unit (AU) value ≥1 was considered positive for IgG. A sample was classified as positive for neutralizing antibodies if its value was ≥0.3 μg/mL.

Immunoglobulin A was detected using the enzyme-linked immunosorbent assay (ELISA) commercial kit COVID-19 Human IgA Spike-RBD of SARS-CoV-2 from Raybiotech (Cat: IEQ-CoVS1RBD-IgA), following the manufacturer’s instructions. The samples were measured using a Biotek EPOCH 2 plate reader. If the obtained value from the plate reader was below the detection limit for IgA detection, the sample was reanalyzed. If the subsequent result remained the same, that particular result was excluded from the analysis, primarily affecting samples from donors who received the CoronaVac (2x) scheme.

### Statistical analysis

This study calculated the geometric mean and 95% confidence interval (CI) for each antibody and vaccine combination. The geometric mean was chosen because immune data often follow a positively skewed, asymmetric distribution. Using the arithmetic mean could distort the interpretation of the data as a measure of central tendency as it is highly influenced by isolated extreme values (18–20). An analysis of variance (ANOVA) test was performed after the logarithmic transformation of the antibody data to assess whether there were statistically significant differences between antibodies from different vaccine combinations. Post-hoc pairwise comparisons were conducted using Tukey’s Honest Significant Difference (HSD) method in cases where a statistically significant result was obtained. This method utilizes the Studentized range distribution to estimate confidence intervals of mean differences between factor levels. To evaluate the difference in neutralizing antibodies by sex within each vaccine combination, *t*-tests were employed.

The statistical analysis applied a logarithmic transformation to the raw data. This transformation is recommended as it allows the data to be scaled appropriately for analysis. When working with immune data, it is common practice to transform the variable to a logarithmic scale (21–23). The logarithmic transformation is a monotonically increasing function, which ensures that the *p*-values computed after the transformation remain applicable when the data are transformed back to the original scale through exponentiation. Similarly, when the 2-sided 95% confidence bounds are computed after logarithmic transformation and then transformed back to the original scale through exponentiation, they represent valid 2-sided 95% confidence bounds on the geometric mean of the data in the original scale. All statistical tests were evaluated at a significance level of 5%. The statistical analyses and generation of charts were performed using R (v.4.2.3), RStudio (v.2022.12.0.353), and GraphPad Prism 8 software.

## Results

### anti-N IgG against SARS-CoV-2 predominates in recovered non-vaccinated individuals and the CoronaVac-vaccinated population

The *N* protein in the CoronaVac platform is a vaccine against SARS-CoV-2. Therefore, we chose to examine the antibodies targeting the *N* protein. To establish true negative controls, we used pre-pandemic serum samples collected before September 2019. The analysis of the *N* protein was conducted following the procedures outlined in the materials and methods section.

**Figure 1:**
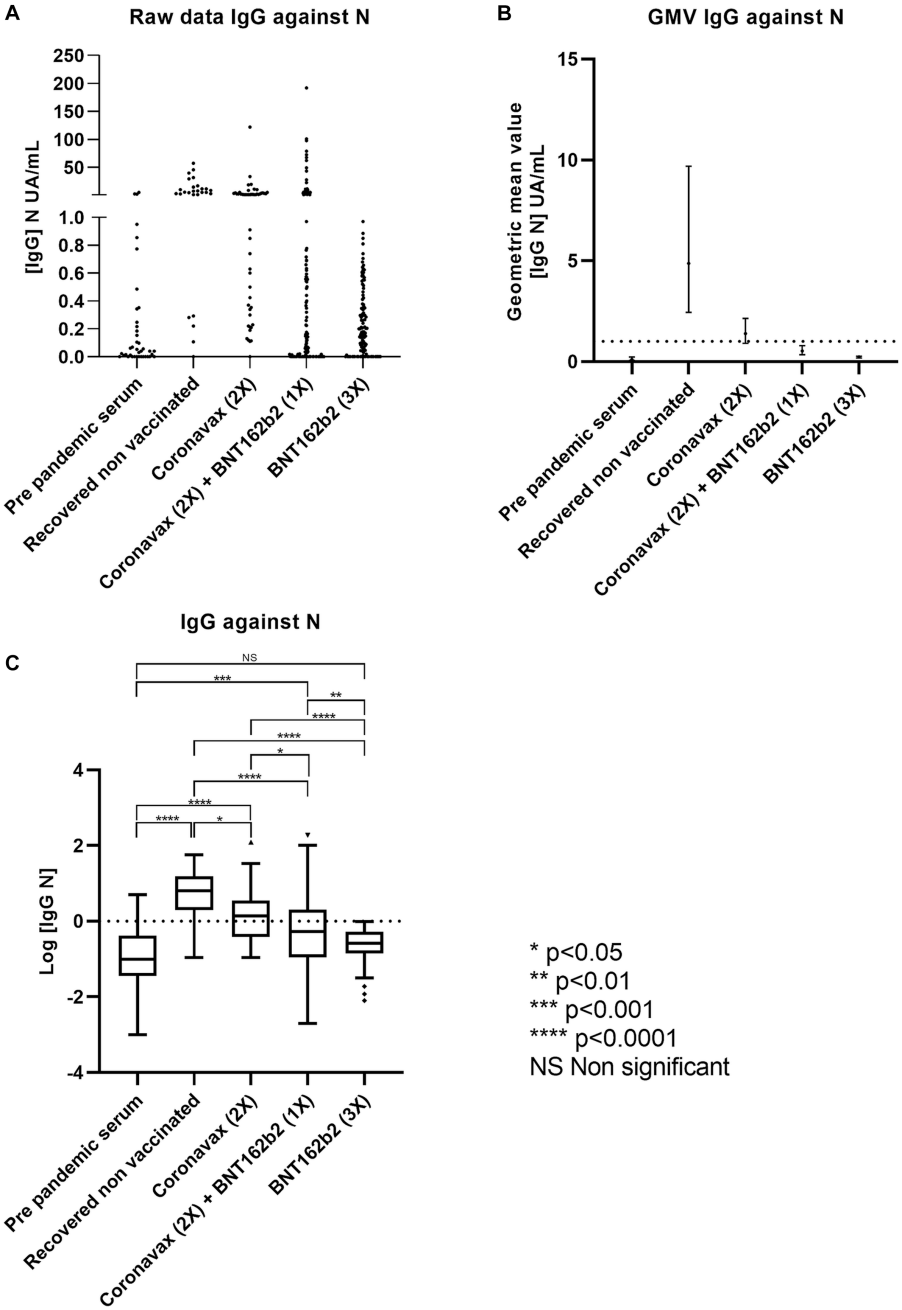
The inactivated virus-based immunization scheme promotes antibodies against the *N* protein of SARS-CoV-2. Serum reactivity against the *N* protein of SARS-CoV-2 was measured by CLIA. Data describes **(A)** raw data, **(B)** GMV with 95% CI, and **(C)** statistical analysis based on Log [IgG-N]. A statistically significant difference between groups was determined with one-way ANOVA (*p* < 0.0001). Significant pairwise differences were obtained by Tukey’s HSD post-hoc (*p < 0.05; **p < 0.01; ***p < 0.001; ****p < 0.001; ns: not statistically significant).

The raw data presented in Figure 1A demonstrate a substantial variation in IgG concentrations across all analyzed groups except for the pre-pandemic serum group. To provide a concise measure of central tendency for immunologic data (19), we calculated the geometric mean value (GMV) to describe the original data in this study. The GMV of IgG against *N* protein concentration in the pre-pandemic serum group was determined to be 0.11 AU (95% CI: 0.05–0.23 AU), whereas the RCN group exhibited a GMV of 4.86 AU (95% CI: 2.44–9.70 AU). Among individuals who received only two doses of CoronaVac, the GMV was 1.39 AU (95% CI: 0.91–2.14 AU). For those vaccinated with the CoronaVac (2x) + BNT162b2 (1x) regimen, the GMV was 0.53 AU (95% CI: 0.35–0.80 AU). Donors who received the BNT162b2 (3x) regimen exhibited a GMV of 0.22 AU (95% CI: 0.19–0.27 AU) (Figure 1B). In this context, any group with a GMV below one was considered negative for the presence of IgG against the *N* protein. We performed a logarithmic transformation of the concentration and conducted one-way ANOVA with Tukey’s HSD post-hoc analysis (if the ANOVA yielded a statistically significant difference) to analyze these data (Figure 1C).

The ANOVA test produced a highly significant result (*p* < 0.0001). As anticipated, we observed that pre-pandemic serum levels of IgG against *N* protein were significantly lower than the vaccination schemes, except for those who received the triple BNT162b2 regimen. Notably, the RCN group exhibited a statistically significant difference in IgG levels compared to the CoronaVac (2x) and CoronaVac (2x) + BNT162b2 (1x) groups. The RCN group displayed the highest concentration of IgG against *N* protein. Among all the vaccination schemes, the CoronaVac (2x) group also exhibited a statistically significant difference when compared to the CoronaVac (2x) + BNT162b2 (1x) and BNT162b2 (3x) groups. Furthermore, the CoronaVac (2x) + BNT162b2 (1x) group demonstrated a statistically significant difference in IgG levels compared to the BNT162b2 (3x) group (Figure 1C). These findings suggest that the CoronaVac (2x)-based vaccination scheme elicits a lower anti-N antibody response to SARS-CoV-2 compared to the group that recovered from COVID-19 and remained unvaccinated.

### anti-S1-RBD levels against SARS-CoV-2 predominate in the homologous and heterologous BNT162b2 vaccinated population

**Figure 2:**
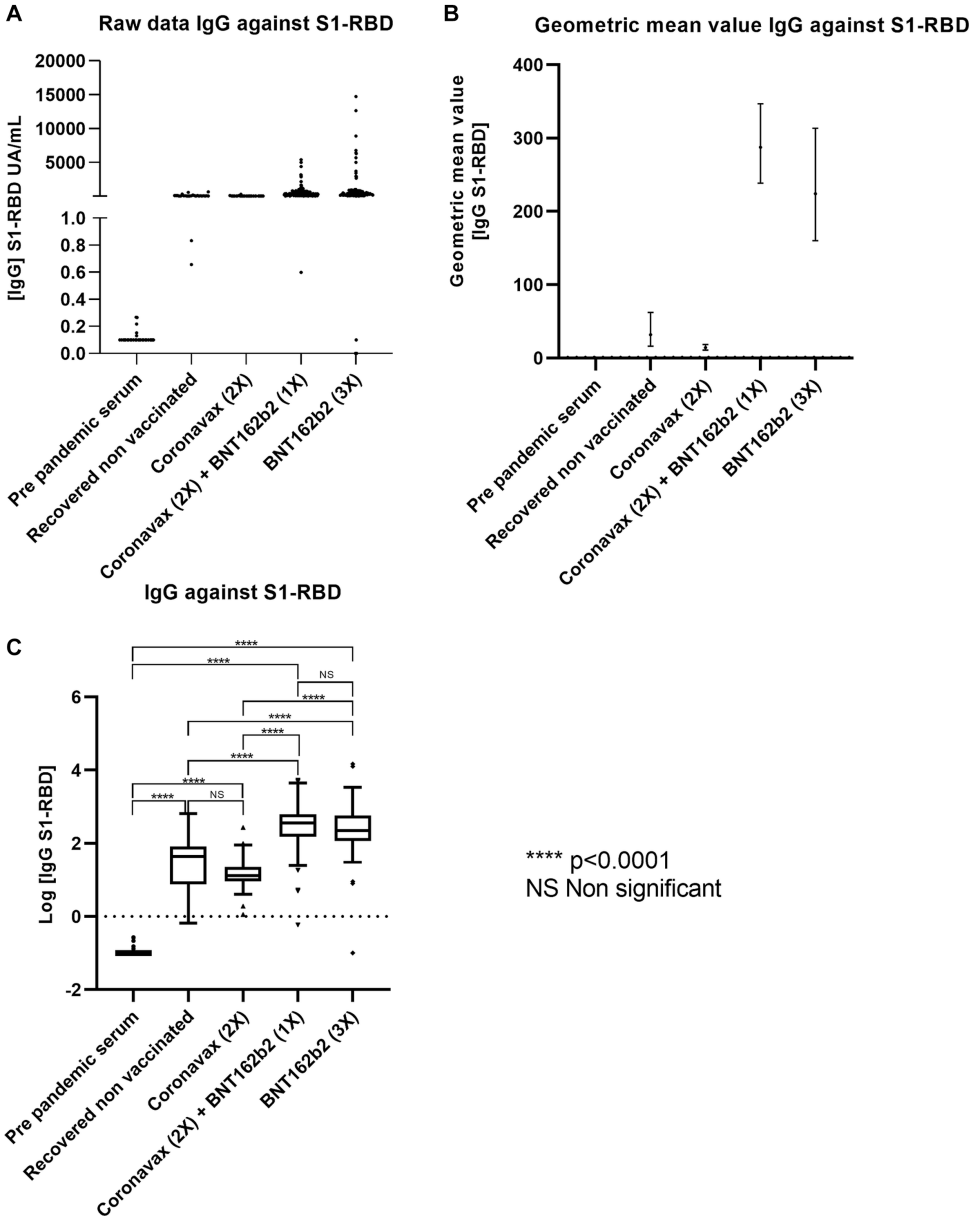
The BNT162b2 mRNA-based scheme promotes antibodies against the RBD-S1 protein of SARS-CoV-2. The Chilean population received different vaccines based on CoronaVac and BNT16b3. Serum reactivity against the S1-RBD protein of SARS-CoV-2 was measured by CLIA. Data describes **(A)** raw data, **(B)** GMV with 95% CI, and **(C)** statistical analysis based on Log [IgG S1-RBD]. A statistically significant difference between groups was determined with one-way ANOVA (*p* < 0.0001). Significant pairwise differences were obtained by Tukey’s HSD post-hoc (****p < 0.001; ns: not statistically significant).

Given the importance of the SARS-CoV-2 S1-RBD protein in the invasion and evasion of the immune response mechanisms, we assessed the IgG levels of anti-S1-RBD antibodies, as outlined in the materials and methods section. The raw data indicate that all the tested conditions, except for the pre-pandemic serum, exhibited positive IgG reactivity against S1-RBD (Figure 2A). The ANOVA test produced a statistically significant result (*p* < 0.0001). Notably, the pre-pandemic serum samples displayed a GMV of 0.11 AU (95% CI: 0.10–0.12 AU). In contrast, the RCN group patients exhibited a GMV of 31.85 AU (95% CI: 16.32–62.14 AU). The GMV for donors vaccinated with CoronaVac (2x) was 14.03 AU (95% CI: 10.73–18.37 AU). For donors vaccinated with CoronaVac (2x) + BNT162b2 (1x), the GMV was 287.6 AU (95% CI: 238.59–346.68 AU), and for those vaccinated with BNT162b2 (3x), the GMV was 199.26 AU (95% CI: 149.58–265.17 AU) (Figure 2B). These findings suggest that the volunteers inoculated with CoronaVac (2x) exhibited the lowest concentration of IgG against S1-RBD.

The concentration data were subjected to logarithmic transformation, following the same procedure described above for IgG against *N* protein (Figure 2C). Our data analysis reveals that only the pre-pandemic serum tested negative for IgG against S1-RBD. Subsequently, we conducted a one-way ANOVA and, upon observing a significant difference, we performed a Tukey multiple comparison test. Comparing the groups, we found no statistically significant difference between the serum samples from the RCN group and those from the CoronaVac (2x) group. Similarly, no statistical difference was observed between the groups of volunteers inoculated with CoronaVac (2x) + BNT162b2 (1x) and BNT162b2 (3x). Notably, all regimens that included a booster with BNT162b2 exhibited higher immunoglobulin concentrations than those inoculated with CoronaVac (2x) alone (Figure 2C). These findings suggest that the BNT162b2 booster increases anti-S1-RBD levels, highlighting an enhanced immune response mediated by the boost.

**Figure 3:**
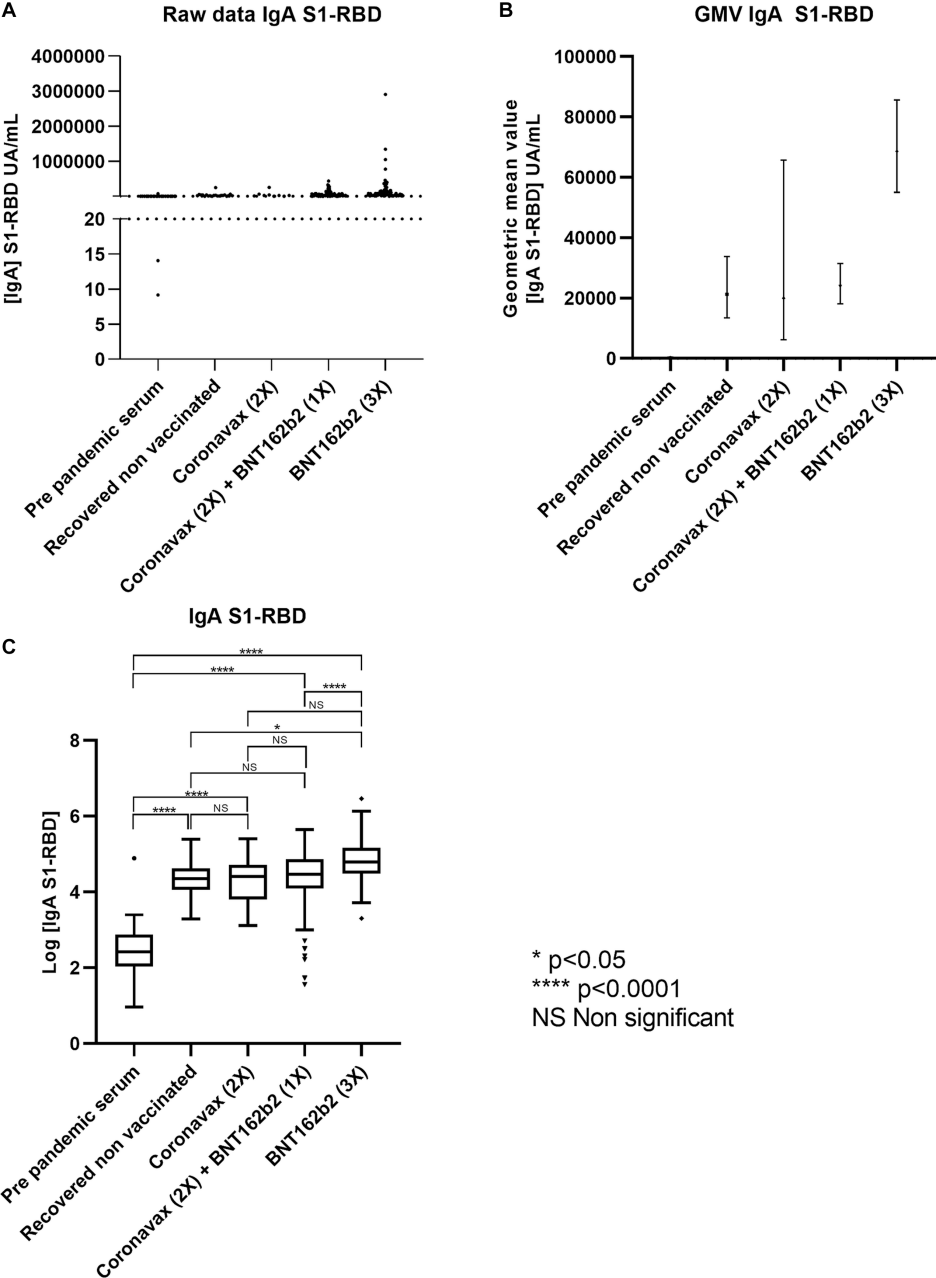
The BNT162b2 mRNA-based scheme promotes IgA antibodies against the RBD-S1 protein of SARS-CoV-2. The Chilean population received different vaccines based on CoronaVac and BNT16b3. Serum reactivity against IgA antibodies to the S1-RBD protein of SARS-CoV-2 was measured by ELISA. Data describes **(A)** raw data, **(B)** GMV with 95% CI, and **(C)** statistical analysis based on Log [IgA S1-RBD]. The statistically significant difference between groups was determined with one-way ANOVA (*p* < 0.0001). Significant pairwise differences were obtained by Tukey’s HSD post-hoc (*p < 0.05; ****p < 0.001; ns: not statistically significant).

We also examined IgA levels against S1-RBD in response to different vaccine combinations. Previous scientific reports have shown a strong correlation between the impact of vaccines on IgA S1-RBD and S1-RBD IgG levels (15, 16). However, some donor samples exhibited IgA levels below the detection limit of the employed technique, necessitating their exclusion from the present analysis. Consequently, the pre-pandemic group comprised 39 out of 40 remaining samples, while the RCN group comprised 21 out of 27. The CoronaVac (2x) group retained 9 out of 53 samples, CoronaVac (2x) + BNT162b2 (1x) retained 157 out of 175 samples, and BNT162b2 (3x) retained 115 out of 128 samples (Figure 3A). The raw data indicate that all analyzed conditions were positive for IgA against S1-RBD (Figure 3A). Specifically, the pre-pandemic serum samples exhibited a GMV of 290.2 AU (95% CI: 174.20–483.45 AU). The RCN group patients showed a GMV of 21,315 AU (95% CI: 13,443.69–33,797.27 AU). For donors vaccinated with CoronaVac (2x), the GMV was 20,265 AU (95% CI: 6,247.30–65,735.70 AU). Donors vaccinated with CoronaVac (2x) + BNT162b2 (1x) had a GMV of 23,871 AU (95% CI: 18,101.87–31,479.83 AU), and those vaccinated with BNT162b2 (3x) exhibited a GMV of 68,664 AU (95% CI: 55,081.83–85,596.05 AU) (Figure 3B).

Notably, vaccination with BNT162b2 (3x) led to the highest increase in IgA against S1-RBD compared to the other groups. Further analysis revealed no statistical difference between the CoronaVac (2x), CoronaVac (2x) + BNT162b2 (1x), and recovered non-vaccinated groups. Additionally, there was no statistical difference between the CoronaVac (2x) and the BNT162b2 (3x) groups. However, the ANOVA test yielded a statistically significant result (*p* < 0.0001), indicating a significant difference between the CoronaVac (2x) + BNT162b2 (1x) group and the BNT162b2 (3x) group, with the latter exhibiting higher IgA levels (Figure 3C). From a statistical standpoint, it is important to interpret the results of CoronaVac (2x) cautiously in comparison to other vaccine combinations due to the following observations: We also analyzed the IgA against S1-RBD stimulated with different vaccine combinations. The scientific report describes a good correlation of vaccine impact between IgA S1-RBD and S1-RBD IgG levels (15, 16). Several donors’ samples exhibited IgA levels that fell below the detection limit of the employed technique, thereby warranting their exclusion from the present analysis. Consequently, the pre-pandemic group comprised 39 out of 40 remaining samples, while the RCN group consisted of 21 out of 27, CoronaVac (2x) retained 9 out of 53, CoronaVac (2x) + BNT162b2 (1x) retained 157 out of 175, and BNT162b2 (3x) retained 115 out of 128 (Figure 3A). The raw data suggest that all the analyzed conditions were positive for IgA against S1-RBD (Figure 3A). Pre-pandemic serum samples showed a GMV of 290.2 AU (95% CI: 174.20–483.45 AU). Patients from the group that recovered from COVID-19 and was not vaccinated showed a GMV of 21,315 AU (95% CI: 13,443.69–33,797.27 AU). The donors vaccinated with CoronaVac (2x) showed a GMV of 20,265 AU (95% CI: 6,247.30–65,735.70 AU). Donors vaccinated with CoronaVac (2x) + BNT162b2 (1x) showed a GMV of 23,871 AU (95% CI: 18,101.87–31,479.83 AU) and those vaccinated with BNT162b2 (3x) showed a GMV of 68,664 AU (95% CI: 55,081.83–85,596.05 AU) (Figure 3B). In this context, vaccination with BNT162b2 (3x) generated the highest increase in IgA against S1-RBD versus the other groups. Further analysis indicates that there was no statistical difference between the donors that received the schemes CoronaVac (2x) and CoronaVac (2x) + BNT162b2 (1x), as well as the recovered non-vaccinated. Also, the group of CoronaVac (2x) compared with BNT162b2 (3x) did not have a statistical difference. However, the ANOVA test yielded a statistically significant result (*p* < 0.0001), where the group of CoronaVac (2x) + BNT162b2 (1x) compared with BNT162b2 (3x) was statistically different in favor of BNT162b2 (3x) (Figure 3C).

From a statistical point of view, CoronaVac (2x) results from the proposed parametric statistical methodology are to be interpreted with caution when compared to other vaccine combinations; we have issued the following observations: (1) the sample size for this group was small after restricting to donors’ serum samples above the detection limit of the technique (*n* = 9). Hence, GMV 95% CIs were wide for this particular group. (2) A wider confidence interval covered a broader area and overlapped other estimated vaccine combination confidence intervals. Hence, corresponding pairwise comparisons were statistically not significant. (3) The adverse effect of non-normality is greater with greater departures from normality, but the effect is relatively small if sample sizes are equal or unequal but large (20) (in this case, a Shapiro–Wilk normality test for log-transformed data yielded statistically significant results for two groups suggesting non-normality; also, unequal and small sample size for one group is observed). (4) An alternative non-parametric statistical test was not considered in the main analysis in order to standardize the analytical and statistical procedure. Also, these tests are known to be less robust (sources argue that most parametric statistical tests can still provide reasonably reliable information with regard to the underlying population even if certain assumptions are violated (24), whereas a parametric method will typically have a lower probability of committing a Type II error) (20). Nevertheless, relying on statistical results derived from confidence intervals or parametric tests considering such a small sample for one group, violating normality and variance assumptions (as for some groups in this case) would not responsibly and substantially contribute to overall immune response research. Therefore, non-parametric results are reported in the next paragraph.

A Kruskal-Wallis test, serving as the non-parametric equivalent of the ANOVA test, was employed to compare the vaccine combinations. Pairwise comparisons were conducted using the Dwass-Steel-Critchlow-Fligner two-sided all-treatments multiple comparisons post-hoc test based on rankings. For the IgA against S1-RBD data, the Kruskal-Wallis test yielded a statistically significant result (*p* < 0.0001), and the pairwise post-hoc test produced similar results to its parametric equivalent. Considering the dispersion of the data and aiming for a more precise interpretation, we focused on the tendencies observed in the GMV of IgA among the groups. The data indicate that donors inoculated with CoronaVac (2x) had a GMV approximately 3.38 times lower than those vaccinated with BNT162b2, 0.17 times lower than those vaccinated with CoronaVac (2x) + BNT162b2 (1x), and 0.05 times lower than the recovered non-vaccinated group. These results, both for IgG and IgA, underscore the enhanced immune response conferred by mRNA-based technology.

### Neutralization by SARS-CoV-2 predominates in homologous and heterologous BNT162b2 vaccinated populations

**Figure 4:**
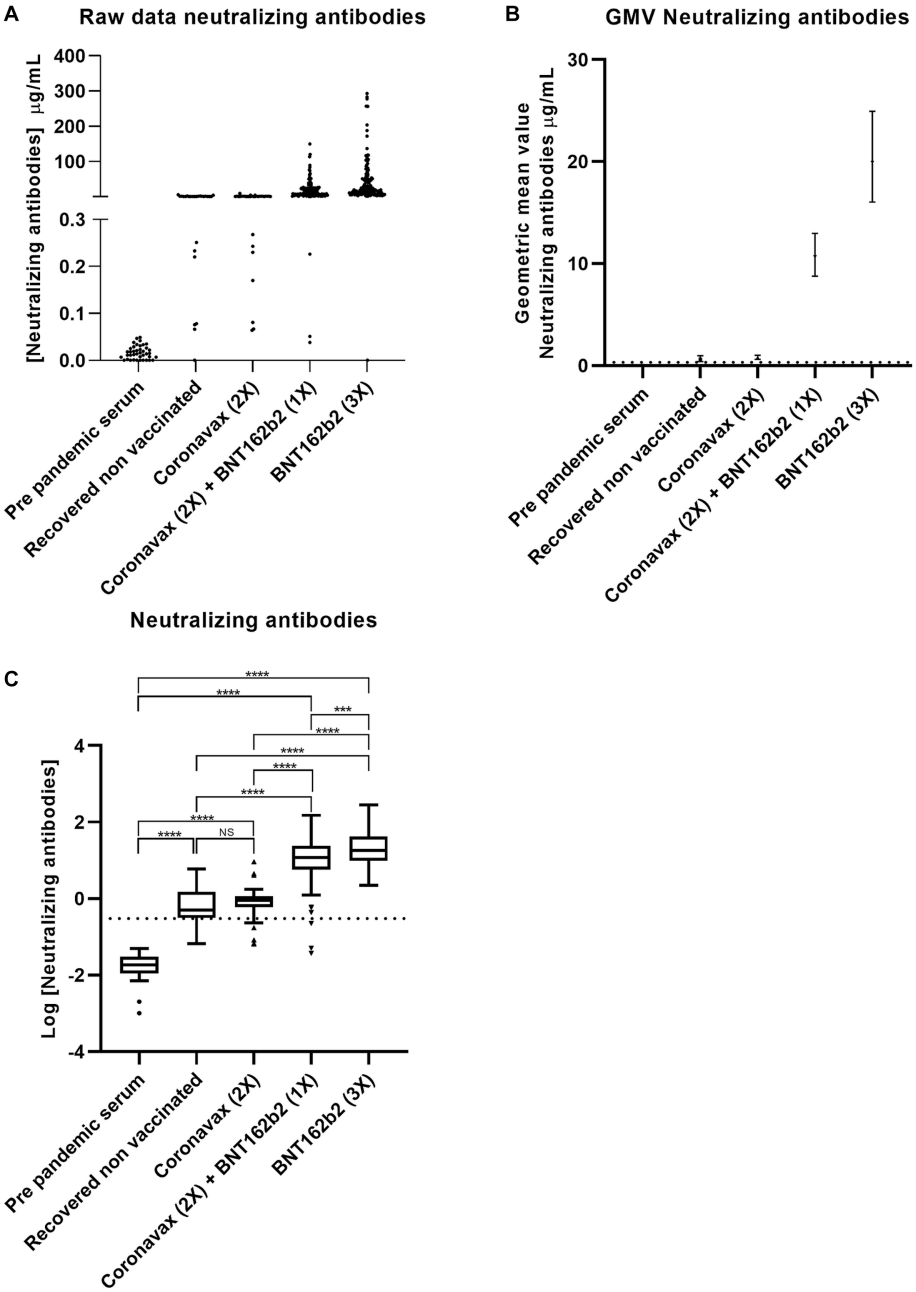
The BNT162b2 mRNA-based scheme promotes neutralization against SARS-CoV-2. The Chilean population received different vaccines based on CoronaVac and BNT16b3. Serum neutralization against SARS-CoV-2 was measured by CLIA. Data describes **(A)** raw data, **(B)** geometric mean values, and **(C)** statistical analysis based on Log [Neutralization antibodies]. Statistical significance was determined using one-way ANOVA (*p* < 0.0001). Significant pairwise differences were obtained by Tukey’s HSD post-hoc (***p < 0.001; ****p < 0.001; ns: not statistically significant).

Given the correlation between antibodies against S1-RBD and vaccine protection against SARS-CoV-2, we aimed to analyze the protective efficacy of sera from different vaccination schemes administered in Chile (25, 26). The CLIA-based methodology employed in this study has demonstrated a strong correlation with plaque reduction neutralization tests (27–29). We quantified neutralizing antibodies using a CLIA-based neutralization assay (27–29). The raw data indicates that all groups under investigation, except for the pre-pandemic serum, tested positive for neutralizing antibodies (Figure 4A). The ANOVA test yielded a statistically significant result (*p* < 0.0001). Samples from the pre-pandemic serum exhibited a GMV of 0.02 μg/mL (95% CI: 0.01–0.021 μg/mL), indicating a lack of neutralizing antibodies. Donors who had recovered from the RCN group presented a GMV of 0.60 μg/mL (95% CI: 0.38–0.96 μg/mL). Samples from donors inoculated with CoronaVac (2x) showed a GMV of 0.79 μg/mL (95% CI: 0.61–1.01 μg/mL), which is relatively low (the cutoff is 0.3 μg/mL) but still considered a positive response for neutralizing antibodies. Samples from donors inoculated with the CoronaVac (2x) + BNT162b2 (1x) scheme exhibited a GMV of 10.67 μg/mL (95% CI: 8.77–12.96 μg/mL), while those inoculated with the BNT162b2 (3x) scheme had a concentration of 18.64 μg/mL (95% CI: 15.46–22.48 μg/mL) (Figure 4B). The neutralizing response was higher and directly associated with applying a booster using BNT162b2. Data analysis involved the logarithmic transformation of the concentration values, followed by a one-way ANOVA. Upon observing a significant difference, a Tukey multiple comparison test was conducted. Only the pre-pandemic serum consistently tested negative for neutralizing antibodies, while all other datasets tested positive. Comparisons between the RCN group and the volunteers inoculated with CoronaVac (2x) did not yield statistical significance. However, both of these groups showed statistical differences when compared to the CoronaVac (2x) + BNT162b2 (1x) and BNT162b2 (3x) groups, with the latter demonstrating a significantly higher neutralizing response (*p* ≤ 0.001) (Figure 4C). Consistent with the levels of antibodies against S1-RBD, BNT162b2-based vaccination increased neutralization against SARS-CoV-2, suggesting that the booster utilizing this technology effectively controlled the pandemic in Chile.

### Immunity induced by CoronaVac and BNT162b2 is independent of gender and body mass index in the Chilean population

**Figure 5:**
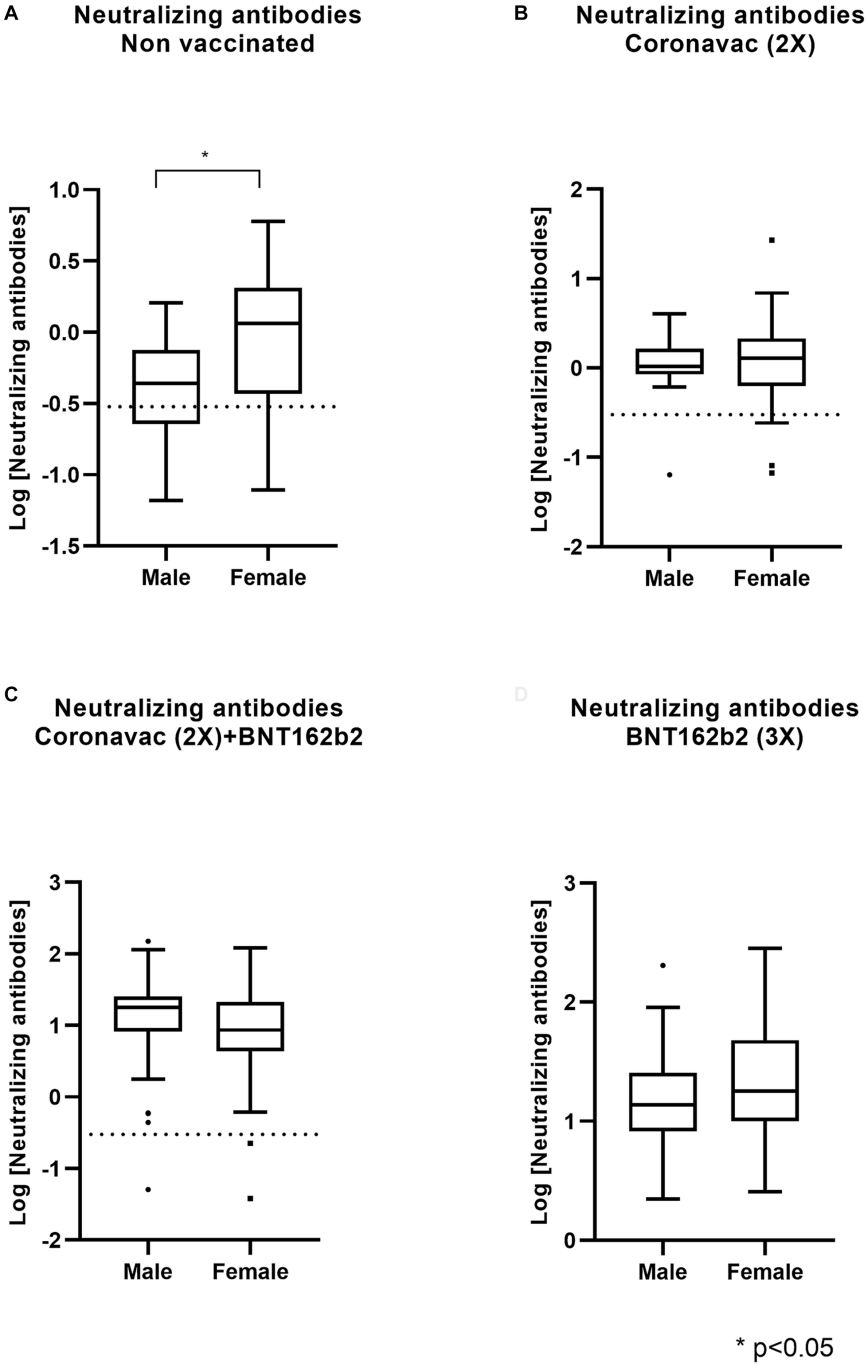
The BNT162b2 mRNA-based scheme promotes neutralization against SARS-CoV-2 equally in men and women. The Chilean population received different vaccines based on CoronaVac and BNT16b3. Recovered from COVID-19 and non-vaccinated participants were used as controls. Serum neutralization against SARS-CoV-2 was measured by CLIA. Data describe statistical contrasts based on Log [Neutralization antibodies] of male and female participants in **(A)** Recovered non-vaccinated group, **(B)** CoronaVac (2x), **(C)** CoronaVac 2x + BNT162b2 (Pfizer), and **(D)** BNT162b2 (3x). Statistical significance was determined using an independent sample *t*-test (ns, not statistically significant; *p < 0.05).

Considering the significance of immune response heterogeneity induced by vaccines, we investigated the impact of gender on the protective immune response against SARS-CoV-2. The demographic and clinical characteristics of the individuals enrolled in this study are outlined in Table 1. An independent sample *t*-test, assuming equal variances (homogeneity assessed with Levene’s test), was employed to compare neutralizing antibody levels between male and female participants. Despite variations in neutralizing antibody levels against SARS-CoV-2, no substantial differences were observed between male and female populations (Figures 5A–D). Notably, a statistically significant result was obtained for the CoronaVac (2x) + BNT162b2 (1x) group (*p* = 0.048). However, a more conservative interpretation was adopted, considering the clinical relevance and acknowledging that the estimated value of *p* is close to the arbitrary 5% threshold (Figures 5A–D).

**Figure 6:**
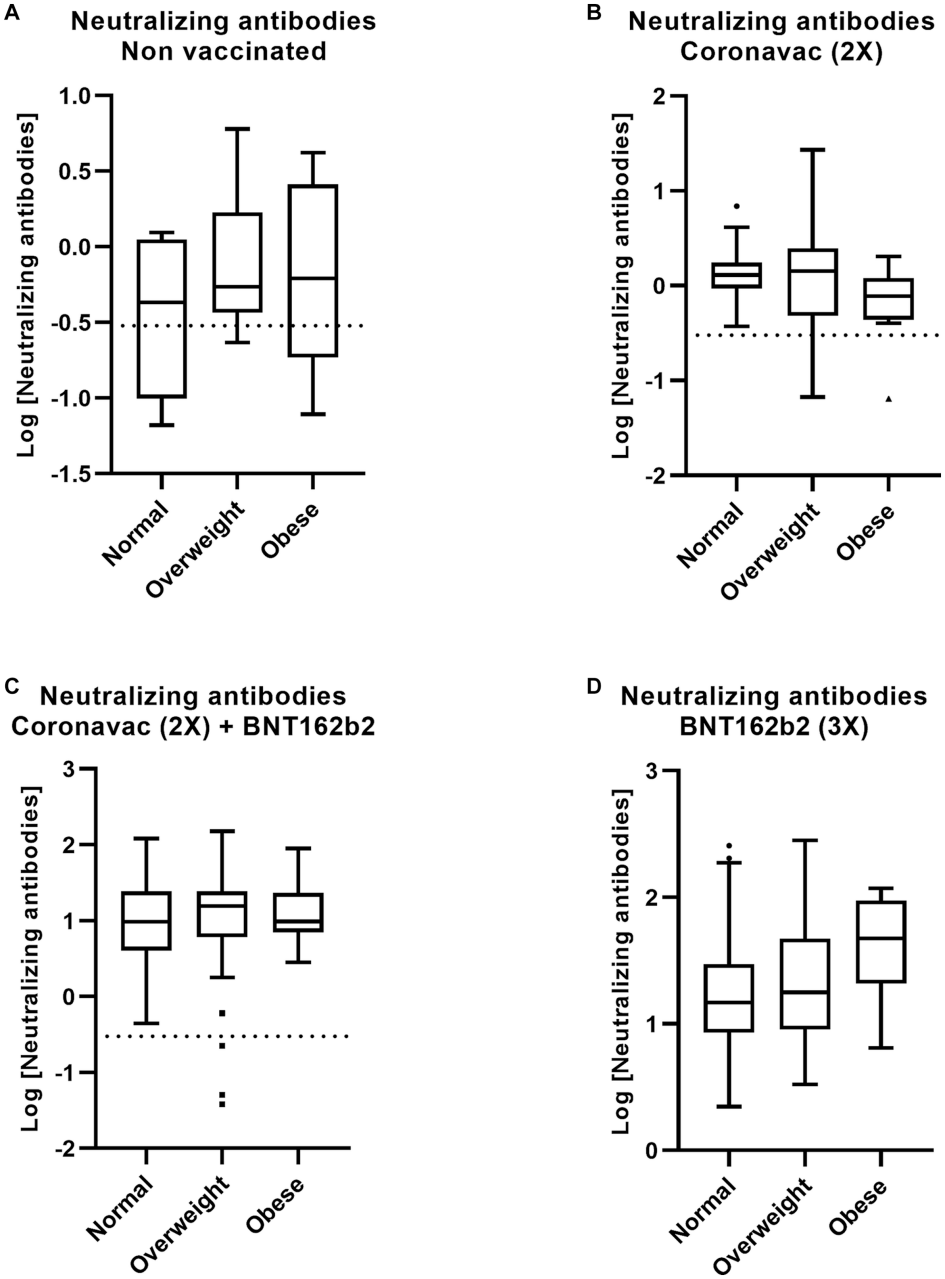
The different vaccination schedules evoke similar immune responses among normal, overweight, and obese people. The Chilean population received different vaccines based on CoronaVac and BNT16b3. Recovered from COVID-19 and non-vaccinated participants were used as controls. Serum neutralization against SARS-CoV-2 was measured by CLIA. The data describe statistical contrasts based on Log [Neutralization antibodies] of normal, overweight, and obesity in **(A)** recovered non-vaccinated population, and with vaccination schemes based on **(B)** CoronaVac (2x), **(C)** CoronaVac 2x + BNT162b2 (Pfizer), **(D)** BNT162b2 (3x). Statistical significance was determined using one-way ANOVA (ns, not statistically significant). Significant pairwise differences were obtained by Tukey’s HSD post-hoc.

Using the volunteers’ weight and height data, we calculated the body mass index (BMI) and classified them as normal, overweight, or obese based on WHO classification. Subsequently, we assessed whether BMI impacted the concentration of neutralizing antibodies within each vaccination scheme. Our analysis revealed no statistically significant association (Figures 6A–D). These findings suggest that gender and BMI do not significantly influence the promotion of a neutralizing immune response against SARS-CoV-2 when employing different vaccination schemes in the Chilean population.

## Discussion

Vaccination can help prevent the overloading of health systems. However, despite global vaccination campaigns (17, 30), the number of cases continues to increase. Fortunately, several safe and effective vaccines have been developed to reduce the risk of infection, severe disease, and death. Examples include BNT162b2 (Pfizer/BioNTech) and mRNA-1,273 (Moderna), among others (8, 31).

Unfortunately, new variants of concern (VOCs) have emerged over time, with varying levels of increased transmissibility and resistance to existing immunity (32–34). These variants have spread widely before eventually declining. Studies have indicated that the antibody responses elicited by CoronaVac and BNT162b2 have decreased after 6 months, possibly contributing to an increase in breakthrough infections (13, 35, 36).

In this study, we utilized the CLIA methodology, which demonstrated excellent concordance with Plaque Reduction Neutralization Tests (PRNT), to propose a potential solution for COVID-19 antibody testing (27–29). We evaluated the humoral immune response of individuals in Chile who received different vaccination schemes, including CoronaVac (2x), CoronaVac (2x) + BNT162b2 (1x), and BNT162b2 (3x). It is important to note that individuals who received CoronaVac (2x) + BNT162b2 (1x) and BNT162b2 (3x) achieved similar S1-RBD antibody titers. Additionally, the group that received at least one BNT162b2 booster exhibited higher neutralization titers against SARS-CoV-2 compared to the pre-pandemic group, the CoronaVac (2x) immunized group, and the RCN group.

Current COVID-19 vaccines primarily target the viral S protein or its receptor binding domain, aiming to induce a robust neutralizing antibody response. However, new vaccines are being developed using the nucleoprotein as an immunogen, which offers broader protection against VOCs at the preclinical stage (37). Individuals who recovered from illness caused by the 2003 SARS-CoV infection have demonstrated long-term memory T cells (lasting 17 years) that are reactive to the SARS-CoV *N* protein and cross-reactive with the *N* protein of SARS-CoV-2 (38).

The inclusion of the S1, M, and *N* proteins in vaccine designs could be considered as potential candidates given their role in activating CD4+ T lymphocytes in patients with mild to moderate COVID-19 (39). Vaccination regimens based on inactivated virus (CoronaVac) and the mRNA vaccine BTN162b2 as a booster have yielded longitudinal study data indicating that infected individuals generated mild neutralizing antibody (nAb) and anti-N IgG titers that declined after 9 months (13, 21). Additionally, immunization of previously unexposed individuals with CoronaVac (2x) resulted in lower nAb titers than convalescent patients, similar to vaccination with a single dose of BTN162b2 (13).

However, our findings align with previous studies. Antibodies against the *N* protein remain slightly positive 3 months after the last immunization in the CoronaVac (2x) group, with a GMV of 1.39 AU and a cutoff for positivity set at one. However, this concentration diminishes over time as the CoronaVac (2x) + BNT162b2 (1x) group shows no presence of IgG against *N* (GMV of 0.53). Furthermore, the group of individuals who had recovered from COVID-19 and were not vaccinated (RCN) exhibited the highest levels of IgG anti-N antibodies. It has been observed that anti-N antibody levels were higher in severe COVID-19 patients compared to those with mild symptoms (40). Although anti-N antibodies were associated with prolonged symptoms after COVID-19 infection, higher levels of IgG anti-N in the first week of SARS-CoV-2 infection were linked to a shorter time to sustained symptom resolution (41). These findings suggest that using inactivated viruses in designing vaccines against SARS-CoV-2 may not be adequate when a durable response against the *N* protein of SARS-CoV-2 is required (37, 42).

Heterologous primary vaccination using CoronaVac (2x) followed by a BNT162b2 booster induces high levels of virus-specific antibodies and potent neutralizing activity against both the ancestral virus and the Delta variant, comparable to the titers obtained after two doses of mRNA vaccines. However, the Omicron variant can evade neutralizing antibodies generated by two doses of mRNA vaccines or CoronaVac (2x) (21). Nevertheless, in the presence of a SARS-CoV-2 infection, one dose of BNT162b2 or two doses of CoronaVac can lead to detectable serum-neutralizing antibodies against Omicron (43). Booster vaccination strategies are crucial, especially considering the decline of neutralizing antibodies over time, particularly in cases where the CoronaVac vaccine initially elicits low neutralizing antibody titers (44).

Our findings align with the aforementioned observations, as the BNT162b2 booster results in an increase in IgG and IgA S1-RBD antibodies compared to CoronaVac (2x), including individuals who have recovered from COVID-19 but were not vaccinated and pre-pandemic sera (15, 16). However, despite the enhanced neutralization seen with CoronaVac (2x) + BNT162b2 (1x), the efficacy of these antibodies in neutralizing the virus differs from that of BNT162b2 (3x), which demonstrates the highest number of neutralizing antibodies among all the study groups. In this case, the antibody concentrations are almost twice as high as those of CoronaVac (2x) + BNT162b2 (1x). It is important to note that while there are variations in the levels of neutralizing antibodies among the different study groups, the antibody titer required to prevent SARS-CoV-2 infection has not yet been established. Therefore, identifying a protective titer against COVID-19 would represent a significant advancement in developing new vaccines against SARS-CoV-2 (45). One limitation of our study is that determining a correlate of protection necessitates comparing immunological data from various laboratories and clinical trials (46).

Vaccination against SARS-CoV-2 can enhance mucosal IgA responses, which are crucial in providing immunity against the virus (15, 16). Notably, IgA antibodies targeting the Spike protein of SARS-CoV-2 exhibit neutralizing activity against the Omicron variant (47). Studies have shown that 2 weeks after the second dose of CoronaVac vaccination, 78.0% of individuals tested positive for IgA-S1 (48). Individuals who received a second dose of CoronaVac can produce salivary IgG-S1, although to a lesser extent, while IgA-S1 was detected at lower levels (49). Immunization with BNT162b2 results in increased levels of IgA-S1, which then decline after 7 months following the first booster dose (50). It has been observed that the peak levels of IgA-S1 occur on day 42 after the booster dose (51). Double-dose CoronaVac induces lower levels of IgA-S1 compared to BNT162b2. The initial immunization with BNT162b2 leads to an increase in IgA-S1 levels (52). Our data support the notion that the vaccination regimen based on BNT162b2 (3x) promotes the production of anti-IgA-RBD antibodies. However, when used as a booster in the CoronaVac (2x) regimen, it does not significantly increase antibody titers against IgA-RBD compared to the RCN group.

Gender differences in immune responses, both innate and adaptive, have been observed following exposure to immunological stimuli (53). Women tend to develop stronger and faster innate and adaptive immune responses than men, which may contribute to the higher frequency of adverse reactions to vaccines observed in women (54). The BNT162b2 vaccine has demonstrated efficacy of 93.7% in women and 96.4% in men who are 16–55 years old (4). Similar patterns have been observed for CoronaVac. Among women who are 18–59 years old, seropositivity rates ranged from 100 to 86%, while in men, seropositivity rates ranged from 94.3 to 86.0% (9).

Regarding the generation of neutralizing antibodies, we did not observe significant differences between women and men following vaccination with BNT162b2 or CoronaVac. However, heterologous vaccination did show a slight but significant impact in the CoronaVac (2x) + BNT162b2 (1x) regimen, leading to greater production of neutralizing antibodies in men than women. Nonetheless, this difference is of little relevance from a clinical perspective as the value is close to the arbitrary threshold of 5%.

Obesity is a growing global public health concern, and individuals with obesity are at a higher risk of morbidity and mortality from COVID-19 (55, 56). It has been observed that vaccination with CoronaVac results in lower levels of antibodies against the Spike protein in individuals with obesity compared to non-obese individuals (57). On the other hand, vaccination with BNT162b2 has been reported to have no impact on the magnitude of antibody titers against the RBD and neutralizing antibodies in obese individuals (30). Our data indicate that overweight and obesity within the Chilean population do not diminish the immunogenicity of the various vaccination schemes studied here.

Our findings also serve to validate the successful vaccination campaign implemented in Chile. Booster doses, such as BNT162b2, exhibit an enhanced immune response against SARS-CoV-2. Since the end of 2020, the emergence of VOCs has prompted research centers to continuously monitor these variants and notable lineages. Future research should focus on developing a Pan-vaccine that can confer prolonged immunity and protection against multiple variants of SARS-CoV-2 (58–60). Currently, there is apprehension regarding the potential of VOCs to evade the immune response elicited by approved vaccines. The suppression of viral replication through vaccination, coupled with the equitable distribution of vaccines, will be crucial in mitigating the risk of these variants.

## Data availability statement

The raw data supporting the conclusions of this article will be made available by the authors, without undue reservation.

## Ethics statement

The studies involving human participants were reviewed and approved by Scientific Ethics Committee of the Health Service of Concepción. Scientific Ethics Committee from the East Metropolitan Health Service Scientific Ethics Committee from Clínica Santa María. The patients/participants provided their written informed consent to participate in this study.

## Author contributions

AR, PD, DD-D, GB, LoA, JG, BR-R, LuA, DFE, DS, CPC, LL, DC, MU, FZ, EN-L, and AV performed the collection and processing of samples. AR, DD-D, GB, CC, LL, VO, and RB assisted in the volunteers’ identification, enrollment, collection of epidemiological, and clinical data. DD-D, LoA, PD, BR-R, LuA, and JG performed SARS-CoV-2-specific antibody ELISAs and the neutralization assays. RP and PD statistical analysis. DD-D, PD, VV-S, GB, MV, and AV writing and editing of the manuscript. CC, JD, LL, RB, MV, HG-E, and AV Planning and logistics. All authors contributed to the article and approved the submitted version.

## Funding

This study was financed and supported by the Public Health Institute of Chile and the Ministry of Science, Technology, Knowledge, and Innovation (project code: ANDID_FI_AVASQUEZ_171). Fellowships were awarded to DD-D (ANID N° 21200880), BR-R (ANID N°21231103), VV-S (ANID N°21232039), DFE (ANID N°21230807), and JG (ANID N°21230817).

## Conflict of interest

The authors declare that the research was conducted in the absence of any commercial or financial relationships that could be construed as a potential conflict of interest.

## Publisher’s note

All claims expressed in this article are solely those of the authors and do not necessarily represent those of their affiliated organizations, or those of the publisher, the editors and the reviewers. Any product that may be evaluated in this article, or claim that may be made by its manufacturer, is not guaranteed or endorsed by the publisher.
